# Genetic Diversity and Population Structure of Asian and European Common Wheat Accessions Based on Genotyping-By-Sequencing

**DOI:** 10.3389/fgene.2020.580782

**Published:** 2020-09-25

**Authors:** Xiu Yang, Binwen Tan, Haijiao Liu, Wei Zhu, Lili Xu, Yi Wang, Xing Fan, Lina Sha, Haiqin Zhang, Jian Zeng, Dandan Wu, Yunfeng Jiang, Xigui Hu, Guoyue Chen, Yonghong Zhou, Houyang Kang

**Affiliations:** ^1^State Key Laboratory of Crop Gene Exploration and Utilization in Southwest China, Sichuan Agricultural University, Chengdu, China; ^2^Triticeae Research Institute, Sichuan Agricultural University, Chengdu, China; ^3^College of Resources, Sichuan Agricultural University, Chengdu, China; ^4^Center of Wheat Research, Henan Institute of Science and Technology, Xinxiang, China

**Keywords:** genetic diversity, population structure, genotyping-by-sequencing, single nucleotide polymorphisms, common wheat

## Abstract

Obtaining information on the genetic diversity and population structure of germplasm facilitates its use in wheat breeding programs. Recently, with the development of next-generation sequencing technology, genotyping-by-sequencing (GBS) has been used as a high-throughput and cost-effective molecular tool for examination of the genetic diversity of wheat breeding lines. In this study, GBS was used to characterize a population of 180 accessions of common wheat originating from Asia and Europe between the latitudes 30° and 45°N. In total, 24,767 high-quality single-nucleotide polymorphism (SNP) markers were used for analysis of genetic diversity and population structure. The B genome contained the highest number of SNPs, followed by the A and D genomes. The polymorphism information content was in the range of 0.1 to 0.4, with a mean of 0.26. The distribution of SNPs markers on the 21 chromosomes ranged from 243 on chromosome 4D to 2,337 on chromosome 3B. Structure and cluster analyses divided the panel of accessions into two subgroups (G1 and G2). G1 principally consisted of European and partial Asian accessions, and G2 comprised mainly accessions from the Middle East and partial Asia. Molecular analysis of variance showed that the genetic variation was greater within groups (99%) than between groups (1%). Comparison of the two subgroups indicated that G1 and G2 contained a high level of genetic diversity. The genetic diversity of G2 was slightly higher as indicated by the observed heterozygosity (*H*_o_) = 0.23, and unbiased diversity index (*uh*) = 0.34. The present results will not only help breeders to understand the genetic diversity of wheat germplasm on the Eurasian continent between the latitudes of 30° and 45°N, but also provide valuable information for wheat genetic improvement through introgression of novel genetic variation in this region.

## Introduction

Wheat (*Triticum aestivum* L.) is an important staple food crop for more than one-third of the world’s population and provides about 20% of calories consumed by humans (Marcussen et al., 2014; Bhatta et al., 2017). As a result of ongoing population growth and climate change, it has been estimated that wheat production must increase by 50% by 2050 (Grassini et al., 2013; Ray et al., 2013; Marcussen et al., 2014). Thus, it seems that wheat production cannot meet demand. Therefore, a challenge for wheat breeders is to improve the stability of grain production and grain yield to meet the growing demand, and to improve resistance and tolerance to biotic and abiotic stresses (Winfield et al., 2018). Analysis of plant genetic diversity is an important aspect of plant breeding, inheritance, conservation, and evolution (Peterson et al., 2014). However, domestication and strong selection pressure by humans, and the use of modern breeding techniques, have increasingly narrowed the gene pool of wheat (Tanksley and McCouch, 1997; Haudry et al., 2007). Therefore, it is essential to enrich wheat germplasm resources by introducing favorable mutations into the cultivated gene pool (Tanksley and McCouch, 1997; Haudry et al., 2007; Zhang et al., 2017).

Morphological traits and molecular markers are two distinct tools for assessment of genetic diversity. However, molecular markers have gained substantial attention because morphological traits are often influenced by the environment (Huang et al., 2002). A variety of molecular markers have been used to study the genetic diversity of wheat, such as randomly amplified polymorphic DNA (Joshi and Nguyen, 1993), random fragment length polymorphisms (Siedler et al., 1994; Kim and Ward, 2000), amplified fragment length polymorphisms (Barrett and Kidwell, 1998; Burkhamer et al., 1998), sequence-tagged sites (Chen et al., 1994) and inter-sequence simple repeats (Nagaoka and Ogihara, 1997). Single-nucleotide polymorphisms (SNPs) and simple sequence repeats (SSRs) are the most commonly used molecular markers for evaluation of genetic diversity among wheat accessions (Huang et al., 2002; Eltaher et al., 2018). Furthermore, SNPs are not only the most abundant type of polymorphism in animal genomes but also exhibit a large number of sequence variants in plant genomes (Ganal et al., 2009, 2012; Rimbert et al., 2018). At present, SNPs are the marker of choice for plant research and plant breeding, such as analyses of marker–trait association, population structure, genomic selection, quantitative trait loci mapping, and research on plant breeding that particularly requires numerous markers (Kumar et al., 2012). Use of high-throughput sequencing technology to discover a large number of SNPs has proved to be not time-consuming and cost-effective (He et al., 2014). With the rapid development of next-generation sequencing (NGS) technologies, an approach for genotyping-by-sequencing (GBS) have been widely used in plant breeding programs (Elshire et al., 2011). GBS enormously reduces the complexity of a large genomes of species by choosing appropriate restriction enzymes (REs) (Poland et al., 2012a), such as wheat with large and complex genomes. Poland et al. (2012b) developed a GBS protocol using two REs (*Pst*I/*Msp*I), which can reduce complexity to a greater extent and achieve a more unified sequencing library than a one-enzyme protocol (Elshire et al., 2011). GBS has been used for genotyping in an increasing number of crops, such as maize, wheat, barley, rice, potato, and cassava. Romay et al. (2013) genotyped a set of 2,815 maize inbred accessions using 681,257 SNPs and observed that some SNPs were related to known candidate genes, involving kernel color, sweetness, and flowering time. Lam et al. (2010) obtained 205,614 SNPs by resequencing 31 soybean genotypes, which offered a precious genomic resource for soybean breeding programs. The GBS protocol was utilized to analyze genetic diversity of 369 Iranian hexaploid wheat accessions, in which a total of 566,439,207 sequence reads were generated and 133,039 SNPs were identified (Alipour et al., 2017). A set of 38,412 GBS-SNPs were identified after sequencing 365 soft winter wheat varieties and F_5_-derived advanced breeding lines originating from multiple crosses in the Cornell University Wheat Breeding Program using a GBS procedure to analyze genetic diversity (Heslot et al., 2013).

The principal region of common wheat cultivation is located between the latitudes of 30°–60°N and 27°–40°S, mainly concentrated in the 30°–45°N region (Nuttonson, 1955). The 180 common wheat accessions used in the present study were collected from 16 countries between the latitudes of 30° and 45°N. The germplasm in this region not only provides novel sources of resistance to biotic and abiotic stresses, but also can enhance the biodiversity of breeding materials. To allow comparison between geographic origin and genotype data, the accessions were grouped into three broad geographical regions, namely Asia, the Middle East, and Europe. The main purpose of this study was to use GBS to evaluate the genetic diversity of the accessions from 16 countries between the latitudes of 30° and 45°N and, in addition, to explore the genetic relationship and population structure of these accessions from different regions.

## Materials and Methods

### Plant Materials

A total of 180 common wheat accessions from 16 countries situated between the latitudes of 30° and 45°N were used in this study (Supplementary Table 1). The seeds were kindly provided by the Triticeae Research Institute, Sichuan Agricultural University, Sichuan, China, the United States Department of Agriculture–Agricultural Research Service (USDA-ARS)–National Plant Germplasm System, United States, and the Xinjiang Academy of Agricultural Sciences, Xinjiang, China.

### Genotyping-By-Sequencing

Total genomic DNA was extracted from fresh young leaves of approximately 2-week-old seedlings using the Hi-DNAsecure Plant Kit DP350 (TIANGEN, Beijing, China). GBS libraries were constructed following the protocol of Poland et al. (2012b). A total of 180 samples were used for genome sequencing on an Illumina HiSeq PE150 platform. SNP calling was performed using TASSEL v. 5.2.40 (Glaubitz et al., 2014). The GBS analysis pipeline used the default parameters (Glaubitz et al., 2014). Paired-end reads were mapped to “Chinese Spring” reference genome with Burrows-Wheeler Aligner (Version: 0.7.8) (Li and Durbin, 2009). The wheat “Chinese Spring” reference genome assembly made available by the International Wheat Genome Sequencing Consortium (IWGSC; RefSeq V1.0) in 2017 was used. The SNPs were filtered based on the criteria minor allele frequency (MAF) threshold <5% and missing values >10 (Li et al., 2015; Saint et al., 2016; Vikram et al., 2016). The detailed information of SNP scores in each of the 180 accessions are available in Supplementary Table 2.

### Population Structure Analysis

Evolutionary relationships among the 180 wheat accessions were determined using the unweighted pair group method with arithmetic mean (UPGMA) based on genetic distances computed with TASSEL. Dendrograms were constructed using the dendrogram function, and then customized using the dendextend package (Galili, 2015) and circlize package (Gu et al., 2014) in R. Principal component analysis (PCA) was performed based on genetic distances among the lines computed with TASSEL (Bradbury et al., 2007). Principal components (PCs) were generated using the covariance method. Eigenvalues were generated to determine the proportion of variation explained by each PC. The first and second PCs were plotted using R.

The population structure of all accessions was evaluated using the Bayesian model-based clustering method in STRUCTURE 2.3.4 software (Pritchard et al., 2000). The STRUCTURE analysis was run five times, with K ranging from 1 to 10, using the admixture model, with burn-in of 100,000 generations and a Markov chain Monte Carlo of 100,000 generations (Chen et al., 2012; Zori´c et al., 2012). To identify the best fit for the number of clusters (K), the Evanno method was utilized (Evanno et al., 2005) using STRUCTURE HARVESTER software (Earl and vonHoldt, 2012). After selection of the optimal K, membership (the proportion of the population assigned to each cluster), mean population differentiation (*F*_*ST*_), and *H*_e_ (Nei, 1978) were determined for each subpopulation identified. The *F*_*ST*_ value (Nei, 1977) of each subpopulation provides an estimate of the degree of fixation of alleles within the subpopulation. The *H*_e_ (analogs to allelic variation in a random mating population) (Nei, 1978) describes the average distance between individuals within the same population, where values close to 0 indicate that the individuals within the population are genetically identical.

### Statistical Analysis

Basic summary statistics were calculated using PowerMarker 3.25 software, comprising gene diversity (GD), polymorphism information content (PIC), MAF, and observed heterozygosity (*H*_o_) (Liu and Muse, 2005). The SNP distribution on each chromosome was counted with 5 Mb as a step, and all SNPs were mapped to IWGSC RefSeq v1.0. The heat map of SNP distribution was plotted using R. On each chromosome, the SNP markers with a PIC value between 0.21 and 0.33 were selected and a total of 7,461 SNPs were used for AMOVA. The number of subpopulations determined on the basis of a STRUCTURE analysis was used for AMOVA. Genetic indices, consisting of number of alleles (*N*_a_), number of effective alleles (*N*_e_), observed heterozygosity (*H*_o_), diversity index (*h*), unbiased diversity index (*uh*), and Shannon’s information index (*I*) were calculated. The AMOVA and estimation of genetic indices were performed using GeneAlEx 6.41 (Peakall and Smouse, 2006).

## Results

### Chromosome Distribution of SNPs

**FIGURE 1:**
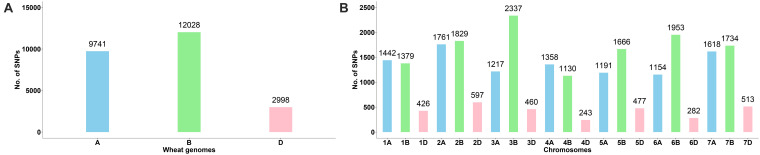
**(A, B)** Chromosomal distribution of SNP markers on all chromosomes in the wheat genomes A, B, and D.

**FIGURE 2:**
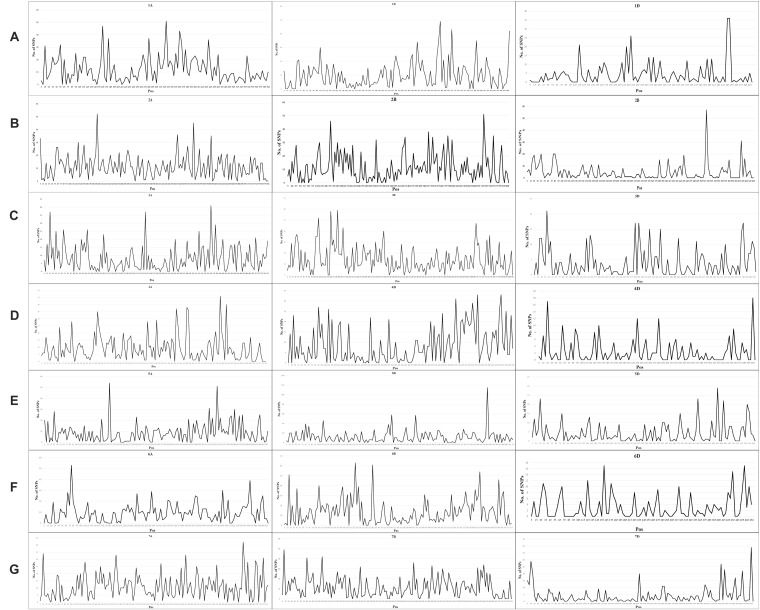
**(A–G)** Heat map of SNPs distribution on each chromosome.

A total of 24,767 SNPs were identified in the A, B, and D genomes. The highest number of SNPs were identified in the B genome (12,028), followed by the A genome (9,741), and the D genome had the lowest number of polymorphic markers with 2,998 (Figure 1A, Supplementary Table 3). In the A genome, chromosome 2A had the highest number of SNPs (1,761), and chromosome 6A harbored the lowest number (1,154); in the B genome, the highest and lowest number of SNPs were detected on chromosomes 3B and 4B (2,337 and 1,130, respectively); in the D genome, chromosome 2D had the highest number of SNPs (597), and chromosome 4D harbored the lowest number (243) (Figure 1B, Supplementary Table 3). The lowest and highest numbers of SNPs identified on an individual chromosome were 243 and 2,337 on chromosomes 4D and 3B, respectively (Figure 1B, Supplementary Table 3). The ratio of number of SNPs in the B to A genomes was 1.23, and that in the B to D and A to D genomes was 4.01 and 3.25, respectively. Thus, the number of SNPs in the A and B genomes exceedingly the number in the D genome, and the number of SNPs in the A genome was only slightly lower than that in the B genome. To characterize the distribution of SNPs in more detail, we used 5 Mb as a step to map all SNPs to the IWGSC RefSeq v1.0, and drew the heat map of SNP distribution on each chromosome (Figure 2). For example, on the 2D chromosome, the physical segment with the highest number of SNPs was 520–525 Mb (Figure 2B). However, the physical segment of 630–635 Mb on the 5B chromosome had the highest number of SNPs (Figure 2E).

### Population Structure

**FIGURE 3:**
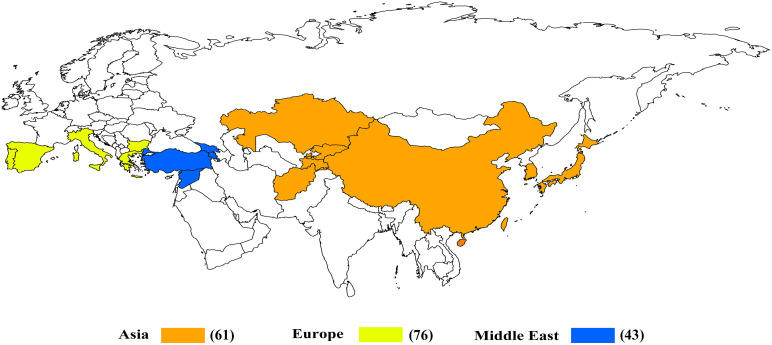
Distribution of common wheat accessions used in this study across Asia and Europe.

**FIGURE 4:**
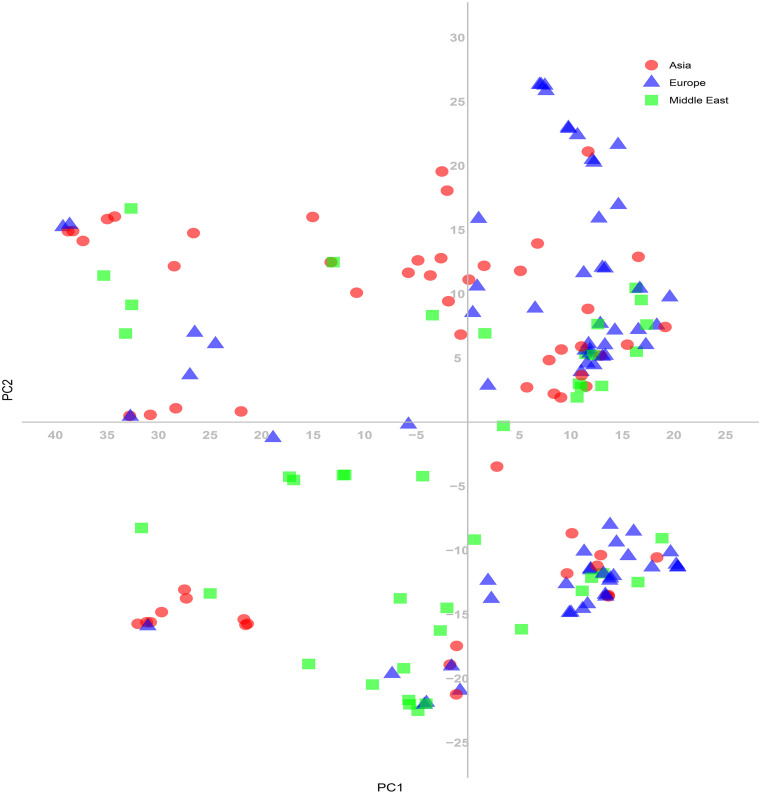
Principal coordinate analysis (PCA) based on genetic distances among the common wheat accessions for the SNP markers.

**FIGURE 5:**
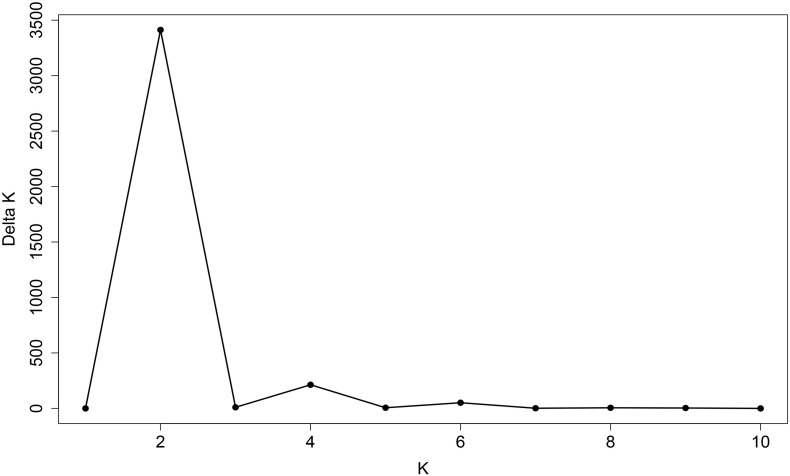
Line graph of delta *K* over *K* from 1 to 10. The highest peak was observed at delta *K* = 2, which suggested the common wheat germplasm comprised two subgroups.

**FIGURE 6:**
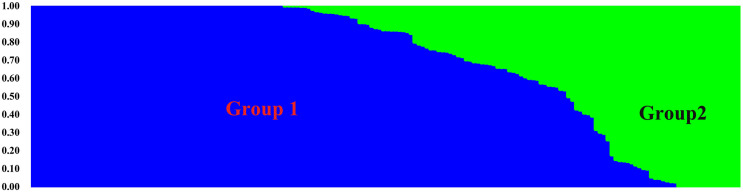
Population structure of 180 common wheat accessions based on 24,767 SNPs markers. The population is divided into two color-coded subgroups. Each bar represents a single accession, and the colored segments within each bar reflect the proportional contributions of each subgroup to that accession.

**FIGURE 7:**
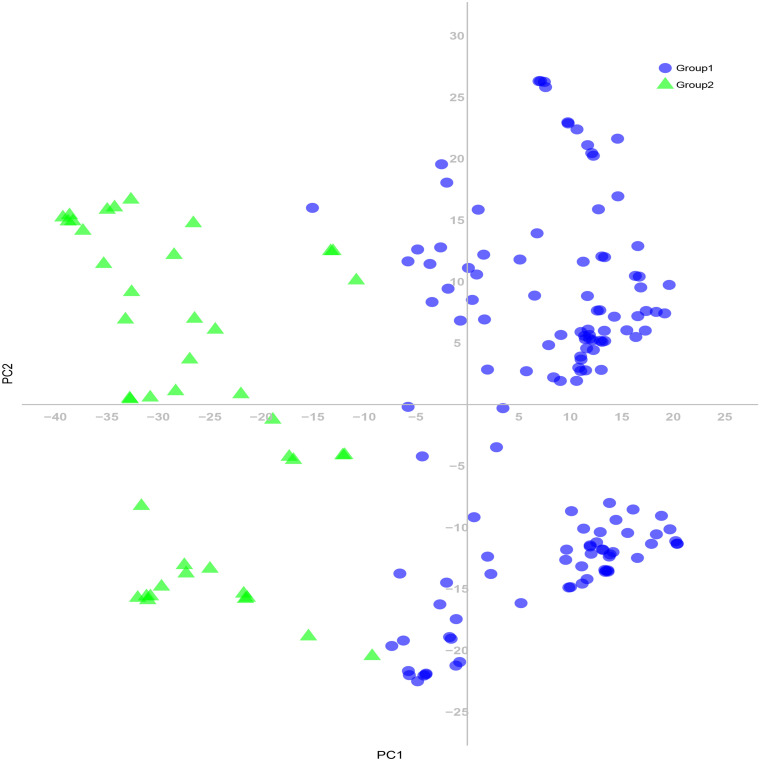
Principal coordinate analysis (PCA) of 180 common wheat accessions based on 24,767 SNP markers. Colors reflect groupings derived from structure analysis.

The 180 common wheat accessions were divided into three broad geographical regions (Figure 3, Table 1). Using PCA, the relationship between the wheat accessions based on the broad geographical regions was determined. In the PCA plot the accessions showed a loose distribution (Figure 4). The accessions from Asia and the Middle East were distributed evenly on PC1 but were less evenly distributed on PC2. The majority of Asian accessions were placed in the positive (upper) portion of the plot. European accessions were mainly clustered towards the right side (positive values) of PC1. And European accessions were divided into two parts by PC2. STRUCTURE analysis was used to study the population structure of the 180 accessions, and delta *K* values obtained were used to determine the optimal number of subpopulations. To determine the optimal *k* value, the number of clusters (*K*) was plotted. At *k* = 2 (Figure 5), a distinct peak was observed, indicating the presence of two subpopulations (Figure 6). Group 1 contained 137 accessions; Group 2 consisted of 43 accessions (Table 2). The degree of genetic differentiation and average distance (*H*_e_) in each subpopulation (Table 2) suggested that the highest degree of genetic differentiation was detected in Group 2 (*F*_st_ = 0.40), whereas the lowest value was observed in Group 1 (*F*_st_ = 0.13). On the other hand, the lowest *H*_e_ was observed in Group 2 and the highest *H*_e_ was detected in Group 1. The results of STRUCTURE analyses (Figure 6), PCA, and the UPGMA cluster analysis (Figure 7, Supplementary Figure 1) showed a high degree of similarity. It was observed that individuals in Group 2 in the STRUCTURE analysis were separated from individuals in Group 1 on PC1. Individual accessions in Group 2 mainly originated from Asia and the Middle East; Group 1 principally consisted of Asian and European accessions. To further understand the clustering relationships among countries, we took the country of origin into consideration. Accessions from Spain were divided into two clusters: one portion was clustered with European accessions, and the other portion clustered with Asian/Middle Eastern accessions. Accessions from Kyrgyzstan, Kazakhstan, China, and Japan tended to cluster with accessions from Europe. Half of the accessions from Afghanistan were clustered with European accessions, and half was grouped with Asian/Middle Eastern accessions. The Middle Eastern accessions originating in Armenia mainly clustered separate from the European accessions.

**TABLE 1:** Provenance of the 180 common wheat accessions used in the study.

**Region**	**Country**	**No. Acc.**
Asia	Afghanistan	12
	Kyrgzstan	4
	Kazakhstan	4
	Tajikistan	4
	China	12
	Korea	15
	Japan	10
Middle East	Turkey	10
	Syria	10
	Georgia	14
	Armenia	9
Europe	Bulgaria	20
	Greece	14
	Italy	17
	Spain	13
	Portugal	12

**TABLE 2:** Results of STRUCTURE analysis of 180 wheat accessions for the fixation index (*F*_st_; indicating significant divergence), average distances (expected heterozygosity), and number of genotypes in each subpopulation.

**Population**	***F*_st_**	**Exp. hetero**	**No. of Genotypes**
G1	0.13	0.31	137
G2	0.40	0.24	43

**FIGURE 8:**
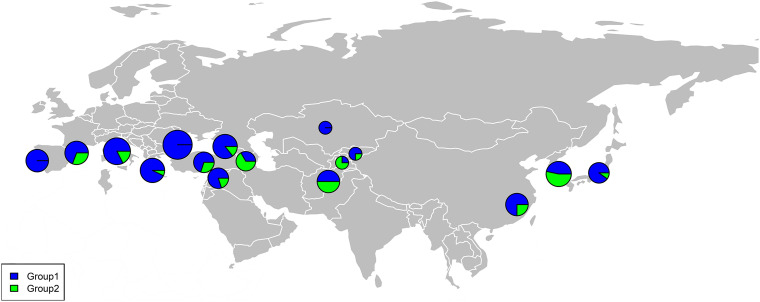
Percentage of each subgroup resolved by STRUCTURE analysis for each country of origin for the common wheat accessions studied.

**FIGURE 9:**
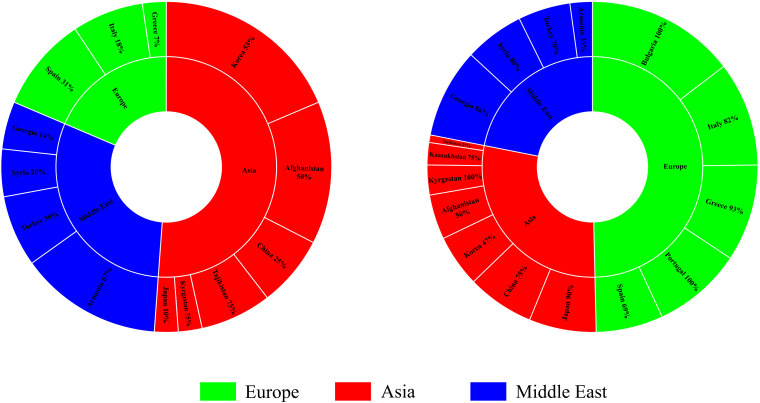
Regions from which the common wheat accessions were collected.

The percentage apportioning to ancestral groups for each country was determined to examine the geographic distribution of the two STRUCTURE groups, which were projected onto a world map (Figure 8). The accessions from Europe were predominantly assigned to Group 1 (blue segments in Figure 8), and the accessions from Bulgaria (100%) and Portugal (100%) were assigned to Group 1 (Figure 9). For the four countries in the Middle East, except for accessions from Armenia (67% accessions in Group 2), the majority of accessions were assigned to Group 1 (Figure 9). Half of the accessions from Afghanistan were assigned to Group 1 and half were assigned to Group 2. In addition, 53% lines from Korea were placed in Group 2 and 47% were placed in Group 1 (Figure 9).

### Genetic Diversity

**FIGURE 10:**
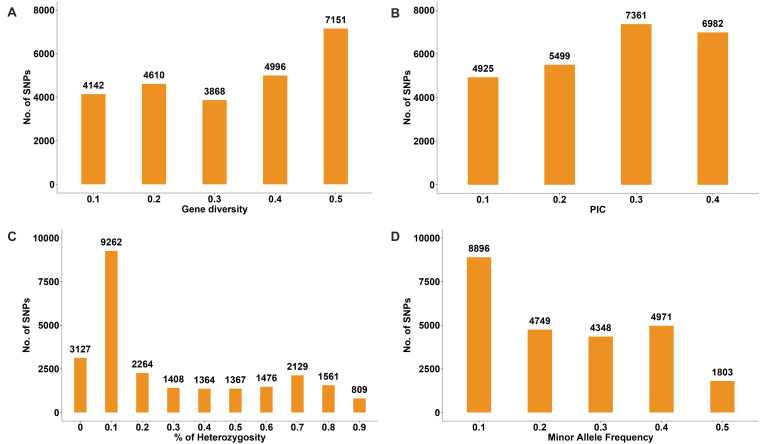
Distribution of genetic diversity **(A)**, polymorphic information content (PIC) **(B)**, percentage of heterozygosity **(C)**, and minor allele frequency **(D)** for 24,767 SNP markers among the 180 common wheat accessions.

The genetic diversity analysis of the 180 accessions revealed that the mean GD and PIC were 0.32 and 0.26, respectively. The GD ranged from 0.1 to 0.5 and PIC ranged from 0.1 to 0.4 (Figures 10A,B). The *H*_o_ values ranged from 0 to 0.9, but for a considerable number of markers the *H*_o_ value was 0.1 (Figure 10C). The average MAF was 0.24 (Figure 10D). Intra-population genetic diversity analysis revealed that mean observed (*N*_a_) and effective (*N*_e_) allele numbers were 2.00 and 1.52 for the two subpopulations, respectively. The value of *N*_e_ in Group 2 (1.52) was higher than that in Group 1 (1.51). The mean values of *I*, *H*_o_, *h*, and *uh* were 0.51, 0.23, 0.33, and 0.34, respectively. However, the Group 2 population showed slightly higher genetic diversity (*H*_o_ = 0.23 and *uh* = 0.34) (Table 3).

**TABLE 3:** Means of genetic parameters for each subpopulation of the 180 wheat accessions. Number of alleles (*N*_a_), number of effective allele (*N*_e_), Shannon’s index (*I*), observed heterozygosity (*H*_o_), diversity index (*h*), and unbiased diversity index (*uh*).

**Pop**	***N*_a_**	***N*_e_**	***I***	***H*_o_**	***h***	***uh***
G1	2.00	1.51	0.51	0.22	0.33	0.33
G2	2.00	1.52	0.51	0.23	0.33	0.34
Mean	2.00	1.52	0.51	0.23	0.33	0.34
						

AMOVA and genetic diversity indices for the two subpopulations were calculated based on the results of the STRUCTURE analysis. The AMOVA revealed much greater variation within populations (99%) than among the populations (1%). High haploid *N*_m_ was observed (28.12), suggesting extremely high gene flow among subpopulations (Table 4). These results revealed low genetic differentiation among the subpopulations, but high genetic differentiation within subpopulations. The UPGMA cluster analysis based on 7,461 markers (Supplementary Figure 2) indicated two subgroups, which was consistent with the population structure analysis (based on 24,767 SNPs).

**TABLE 4:** Analysis of molecular variance using 7461 SNP markers of genetic differentiation among and within two subpopulations of the 180 common wheat accessions.

**Source**	**df**	**SS**	**MS**	**Est. Var.**	**%**	***P* value**
Among Pops	1	5,599.57	5,599.57	31.47	1%	0.001
Within Pops	178	630,097.27	3,539.87	3,539.87	99%	0.001
Total	179	635,696.84		3,571.34	100%	0.001
Nm			28.12			

## Discussion

Wheat germplasm resources are extremely important for breeders. One main wheat-producing area of the world is located between 30° and 45°N latitude, which is rich in wheat germplasm resources. A prerequisite for making full use of these germplasm resources is to assess their genetic diversity (Hawkes, 1981). We used GBS technology to discover a large number of SNPs for genotyping hexaploid wheat derived from diverse provenances in this study. In the present study, we obtained 24,767 SNPs markers and observed the lowest frequency of SNPs in the D genome, whereas the B genome contained the highest frequency of polymorphic markers, which is in agreement with the results of previous studies (Chao et al., 2009; Akhunov et al., 2010; Poland et al., 2012a; Berkman et al., 2013; Würschum et al., 2013; Marcussen et al., 2014; Shavrukov et al., 2014; Edae et al., 2015; Alipour et al., 2017; Eltaher et al., 2018; Rufo et al., 2019). D genome is the youngest one among the three genomes in wheat evolutionary history (Talbert et al., 1998; Caldwell et al., 2004). It is likely that older genomes underwent gene duplication and accumulated more mutations that led to sequence polymorphism (Dvorak et al., 2006). Substantial early gene flow could have occurred between *T. aestivum* and its tetraploid progenitor *T. turgidum* (AABB) but not between the hexaploid and *Aegilops tauschii* (DD) (Caldwell et al., 2004; Dvorak et al., 2006). This could have resulted in greater sequence diversity in the A and B genomes than in D genome (Talbert et al., 1998; Caldwell et al., 2004; Dvorak et al., 2006; Berkman et al., 2013). Furthermore, the fewest SNP markers were located on chromosome 4D, whereas the highest number of SNP markers were located on chromosome 3B, as reported by Saintenac et al. (2013) and Alipour et al. (2017). Eltaher et al. (2018) obtained 25,566 SNPs by GBS for 270 F_3:6_ Nebraska winter wheat accessions, and observed that the highest number of SNPs were located on chromosome 3B, whereas chromosome 3D carried the lowest number of SNPs. Bhatta et al. (2017) reported that chromosomes 2B and 4D had the highest and lowest numbers of SNPs, respectively. Chromosome 4D had the lowest number of markers and chromosome 1B had the highest number of markers in the study by Sukumaran et al. (2015). Allen et al. (2017) used 35,143 SNPs and reported that chromosome 2B had the highest number of markers and chromosome 4D had the lowest number of markers. In contrast, the present study showed that chromosome 3B harbored the highest number of SNPs and chromosome 4D had the lowest number. Meanwhile, we found some SNP hot spot regions in heat map of each chromosome harboring important QTLs. In the 160–170 Mb of chromosome 2B (Figure 2B), Boukhatem et al. (2002) analyzed a set of 98 *F*_8_ recombinant inbred (RI) lines and found a QTL (*QYR-2B.2*) which was associated with yellow rust resistance. Similarly, in the 680–690 Mb of chromosome 1B (Figure 2A), a QTL (*QTgw.ipk-1B-FS4*) which was associated with TKW was identified (Nezhad et al., 2012).

The PIC contributes to a detailed understanding of the level of polymorphism between genotypes. On the basis of previous reports, the PIC can be divided into three categories: (1) when PIC > 0.5, the marker is considered to be highly polymorphic, (2) when 0.25 < PIC < 0.5, the marker is a moderately informative, and (3) when PIC < 0.25, the marker is a low-information marker (Botstein et al., 1980). Lopes et al. (2015) observed a PIC value of 0.27 using the 9K SNP array to genotype the WAMI population, and showed that spring wheat contained moderate levels of polymorphism. Novoselović et al. (2016) genotyped a Croatian panel using a set of 1,229 Diversity Arrays Technology (DArT) markers and obtained an average PIC value of 0.30 among the populations, which indicated that the accessions from Croatia exhibited moderate polymorphism. Eltaher et al. (2018) analyzed 270 F_3:6_ Nebraska winter wheat accessions, and observed a PIC value of 0.25, which indicated that the population contained moderate genetic diversity. El-Esawi et al. (2018) used 1,052 DArT markers to genotype Australian and Belgian wheat accessions, and obtained PIC values of 0.33 and 0.29, respectively, which demonstrated that Australian and Belgian wheat contained moderate genetic diversity. The present results showed that the mean PIC value (0.27) was in agreement with the above-mentioned studies, which indicated that the 180 accessions contained moderate polymorphism. On the other hand, Hao et al. (2011) genotyped 250 Chinese wheat accessions with 512 SSR markers and observed a PIC value of 0.65, which demonstrated that Chinese wheat showed high genetic polymorphism. Zhang et al. (2010) analyzed 205 elite wheat accessions in the United States, using 245 SSR markers, and obtained a PIC value of 0.54, which indicated that the accessions showed a high level of polymorphism. Relative to SSR markers, the lower PIC value of the SNP and DArT markers may be explained by their bi-allelic nature and slow mutation rate (Thuillet et al., 2002; Chesnokov and Artemyeva, 2015).

In the present study, we obtained meaningful information on genetic diversity indices in each subpopulation. High levels of genetic diversity were detected in Groups 1 and 2. The results of AMOVA showed that a high level of genetic diversity was observed within subpopulations, whereas the variation among subpopulations was extremely low (1%). This result may be caused by breeders selecting for specific traits, such as yield, stripe rust resistance, and herbicide tolerance. However, the low genetic variability among subpopulations is explained by the high gene flow (Arora et al., 2014). Wright (1965) showed that when *N*_m_ (haploid) values are less than 1, gene exchange among subpopulations is limited. In the present study we observed an extremely high *N*_m_ value (28.12), indicating that high gene flow led to low genetic differentiation among subpopulations. The results of this study will not only help breeders to understand the genetic diversity of wheat germplasm on the Eurasian continent between the latitudes of 30° and 45°N, but also provides valuable information for genetic improvement of wheat through inclusion of novel genetic variation from China and certain other countries.

The PCA revealed a degree of broad geographic partitioning of the accessions. A previous study by Winfield et al. (2018) used 32,443 polymorphic markers to genotype 804 hexaploid wheat accessions originating from more than 30 countries around the world, and observed that the majority of accessions from Europe clustered together, separated from the majority of Asian and Middle Eastern accessions. Similarly, in the study of Cavanagh et al. (2013), the European winter wheat population showed the strongest degree of genetic differentiation from the remaining populations. Balfourier et al. (2007) used a set of 38 SSR markers to analyze 3,942 accessions originating from 73 countries, and observed that accessions from several Near Eastern and Central Asian areas were grouped in the same subcluster and those from Far Eastern countries clustered together. Strelchenko et al. (2005) analyzed 78 wheat landraces originating from 22 countries and reported that the landraces were separated into European and Asian groups. Chen et al. (2019) reported that West Asian landraces, the majority of European landraces, several South and Central Asian landraces, and the majority of East Asian cultivars clustered together, whereas the majority of East Asian landraces were clustered with several West Asian landraces and the majority of South and Central Asian landraces. Lee et al. (2018) reported that many accessions from Afghanistan, Japan, and Korea were clustered in the same group, while germplasm from China, the Middle East, and Caucasus clustered in a separate group, and an intermediate group largely consisted mainly of accessions from Afghanistan, Japan, and Korea. In the present study, although there was substantial overlap between clusters, the majority of accessions from Europe clustered together, whereas the accessions from Asia and the Middle East were distributed evenly on PC1 (Figure 4). However, the relationships of three overlapping subgroups was unclear, which raises the possibility of exchanging adapted germplasm. To obtain useful information on the genetic diversity and population structure of the accessions, they were divided into two subgroups on the basis of the population structure analysis (Figure 6). In the PCA (Figure 7), genotypes clustered consistent with the subpopulations identified in the STRUCTURE analysis. Moreover, the UPGMA cluster analysis (Supplementary Figure 1) was consistent with the results of the STRUCTURE analysis. The majority of European accessions were divided into Group 1, especially accessions from Bulgaria and Portugal (Figure 9), whereas portions of the Asian and Middle Eastern accessions were distributed in Groups 1 and 2, respectively. The accessions from Turkey, Syria, Georgia, Armenia, Afghanistan, Kyrgyzstan, and Tajikistan showed complex genetic backgrounds, which is not surprising. The area between the Black Sea and the Caspian Sea, and just south of this region (Iraq), is the assumed location of the center of origin of wheat domestication and seems to be a site of population consolidation. Chen et al. (2019) showed that Chinese wheat accessions were mainly derived from European landraces. In the present study, the accessions originating from China tended to cluster with European accessions (Figure 9). The improvement of Chinese wheat was based on hybridization programs, which included well-adapted landraces and introduced accessions. Furthermore, the introduction of foreign materials would promote the genetic improvement of Chinese wheat. Italian varieties including Villa Glori, Mentana, Funo, Abbondanza, St 2422/464, and Libellula were widely cultivated and utilized in the Yellow and Huai River valley winter wheat region, lower and middle Yangtze River valley winter wheat region, southwestern winter wheat region, and northwestern spring wheat region (Zhao et al., 2019). Varieties such as Ukraine 0246, New Ukraine, Red Star, Kavkaz, and Aurora from the former USSR were introduced and disseminated widely in Xinjiang (He et al., 2001). Therefore, the exchange and utilization of germplasm worldwide was an established way to expand the genetic basis of wheat breeding (Zhao et al., 2019).

## Conclusion

In this study, a GBS protocol was used to investigate the population structure and genetic diversity of wheat accessions originating from the Eurasian continent between the latitudes of 30° and 45°N. The panel of 180 accessions was divided into two subgroups, which could be identified by their parentage and selection history. Group 1 principally consisted of European and a portion of Asian accessions, and Group 2 predominantly comprised Middle East and a portion of Asian accessions. Groups 1 and 2 showed high values for genetic diversity indices, which were higher for Group 2. The present results demonstrated that the 180 accessions represent high genetic diversity and can be used for future breeding programs to develop new wheat cultivars with desirable characteristics such as high yield potential, tolerance to biotic and abiotic stress, and good end-use quality, while being well-adapted to diverse environments in China and other countries.

## Data Availability Statement

We have uploaded our SNP sequencing data to FigShare repository (https://figshare.com/s/157a9613b935814cc2d5).

## Author Contributions

HK and YZ conceived and designed the research. XY, BT, and HL conducted the experiments. WZ, LX, YW, XF, LS, JZ, DW, YJ, and XH participated in the preparation of the reagents and materials. HZ and GC analyzed the data. XY and HK wrote the manuscript. All authors read and approved the manuscript.

## Conflict of Interest

The authors declare that the research was conducted in the absence of any commercial or financial relationships that could be construed as a potential conflict of interest.

## Supplementary Material

SUPPLEMENTARY FIGURE 1 UPGMA clustering dendrogram generated using 24,767 SNPs and 180 common wheat accessions. Colors reflect groups derived from STRUCTURE analysis.

SUPPLEMENTARY FIGURE 2 Cluster analysis based on the genetic distances among the 180 accessions for 7,461 SNPs used for AMOVA.

SUPPLEMENTARY TABLE 1 Details of the 180 common wheat accessions used in the study.

SUPPLEMENTARY TABLE 2 The detailed information of SNP scores in each of the 180 accessions.

SUPPLEMENTARY TABLE 3 Chromosomal distribution of the SNP markers used in the study.
