# Supplementary figures and images for: Genetic Diversity and Population Structure of Asian and European Common Wheat Accessions Based on Genotyping-By-Sequencing
Source: Front Genet. 2020 Sep 25;11:580782. doi: 10.3389/fgene.2020.580782 (PMC7545058; doi:10.3389/fgene.2020.580782)

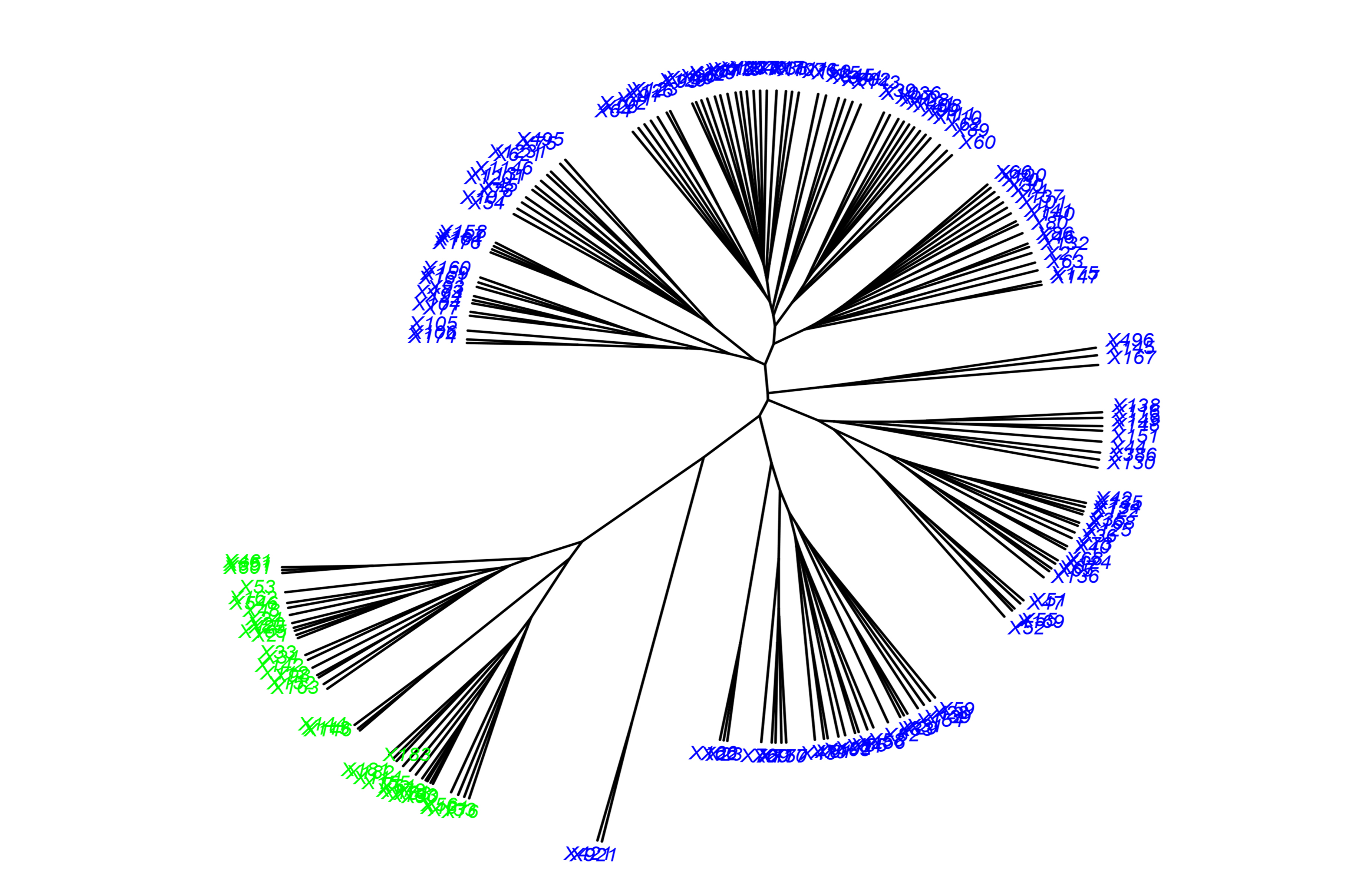

Supplement: SUPPLEMENTARY FIGURE 1 — UPGMA clustering dendrogram generated using 24,767 SNPs and 180 common wheat accessions. Colors reflect groups derived from STRUCTURE analysis. [file Image_1.JPEG]

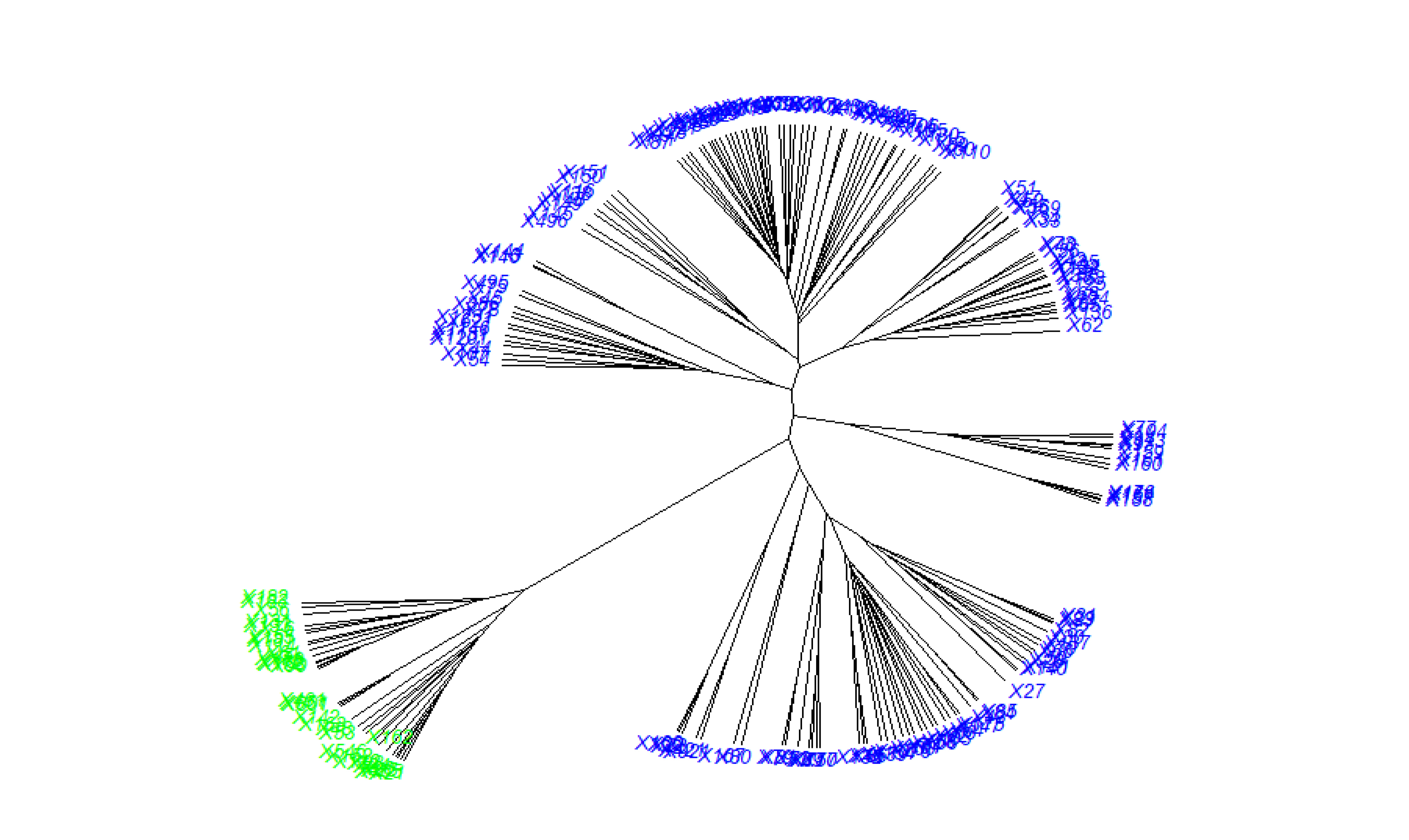

Supplement: SUPPLEMENTARY FIGURE 2 — Cluster analysis based on the genetic distances among the 180 accessions for 7,461 SNPs used for AMOVA. [file Image_2.JPEG]
